# Probing Biomolecular Interactions with Paramagnetic Nuclear Magnetic Resonance Spectroscopy

**DOI:** 10.1002/cbic.202400903

**Published:** 2025-01-13

**Authors:** Hannah Busch, Muhammad Yasir Ateeque, Florian Taube, Thomas Wiegand, Björn Corzilius, Georg Künze

**Affiliations:** ^1^ Institute of Technical and Macromolecular Chemistry RWTH Aachen University Worringerweg 2 52074 Aachen Germany; ^2^ Institute for Drug Discovery University of Leipzig Brüderstr. 34 04103 Leipzig Germany; ^3^ Institute of Chemistry, Department Life, Light & Matter University of Rostock Albert-Einstein-Str. 27 18059 Rostock Germany; ^4^ Max Planck Institute for Chemical Energy Conversion Stiftstr. 34–36 45470 Mühlheim/Ruhr Germany

**Keywords:** Biomolecular interactions, paramagnetic NMR, dynamic nuclear polarization, pseudocontact shifts, paramagnetic relaxation enhancement

## Abstract

Recent advances in computational methods like AlphaFold have transformed structural biology, enabling accurate modeling of protein complexes and driving applications in drug discovery and protein engineering. However, predicting the structure of systems involving weak, transient, or dynamic interactions, or of complexes with disordered regions, remains challenging. Nuclear Magnetic Resonance (NMR) spectroscopy offers atomic‐level insights into biomolecular complexes, even in weakly interacting and dynamic systems. Paramagnetic NMR, in particular, provides long‐range structural restraints, easily exceeding distances over 25 Å, making it ideal for studying large protein complexes. Advances in chemical tools for introducing paramagnetic tags into proteins, combined with progress in electron paramagnetic resonance (EPR) spectroscopy, have enhanced the method's utility. This perspective article discusses paramagnetic NMR approaches for analyzing biomolecular complexes in solution and in the solid state, emphasizing quantities like pseudocontact shifts, residual dipolar couplings, and paramagnetic relaxation enhancements. Additionally, dynamic nuclear polarization offers a promising method to amplify NMR signals of large complexes, even in complex environments. The integration of AlphaFold protein structure prediction with paramagnetic NMR holds great potential for advancing our understanding of biomolecular interactions.

## Graphical abstract

Paramagnetic NMR spectroscopy offers useful opportunities for structural biology. Key techniques such as pseudocontact shifts and paramagnetic relaxation enhancements provide long‐range structural restraints, while dynamic nuclear polarization can amplify NMR signals of large biomolecules in complex environments. The integration of paramagnetic NMR data with computational methods like AlphaFold holds great potential to elucidate large and dynamic biomolecular complexes.

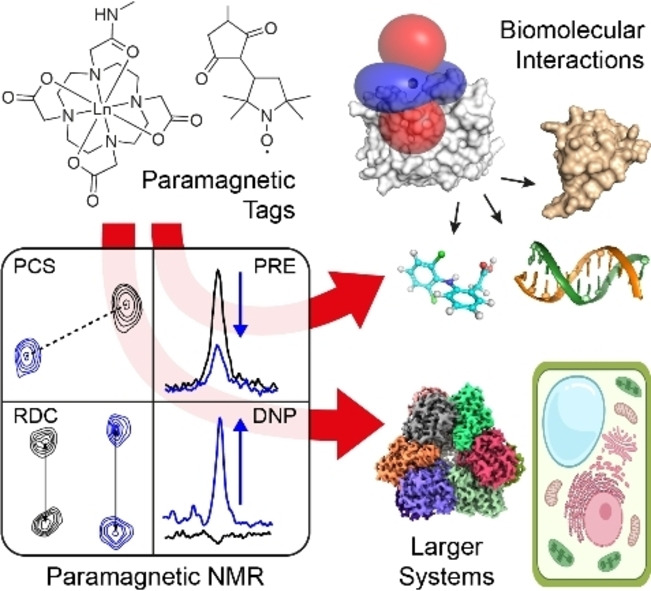

## Introduction

Studying biomolecular interactions is crucial to understand the mechanisms underlying biological processes. Biomolecular interactions govern many cellular functions such as signal transduction, gene regulation, metabolic pathways, and cellular communication. To determine the structures of biomolecular complexes, X‐ray crystallography, NMR spectroscopy and cryo‐electron microscopy (cryo‐EM) are the most frequently employed methods. Successful application of these methods has led to the discovery of over 220.000 structures that were deposited in the Protein Data Bank (PDB) until 2024.^1^ Major advancements in the field of biomolecular structure determination have also come from artificial intelligence methods, such as AlphaFold,^[1]^ RoseTTAFold,^[2]^ and ESMFold,^[3]^ which are able to predict protein structures from amino‐acid sequences with high accuracy. Recently, updated versions of these methods, called AlphaFold3,^[4]^ RoseTTAFold‐All‐Atom,^[5]^ and ESM3,^[6]^ which are able to predict the joint structure of complexes including proteins, nucleic acids, small molecules, ions and post translationally modified residues, were developed. These methods have significantly broadened the scope of biomolecular structure prediction, however, there is still need for improvement in prediction accuracy.

It has to be acknowledged that the conformational space of biomolecular complexes is considerably larger than that of single proteins. While there are approximately 20.000 protein‐coding genes in the human genome, the number of protein‐protein interactions is estimated to be at least 30‐times larger.^[7]^ Certainly, elucidating the numerous and complex interactions that proteins form within the biological environment represents an upcoming significant challenge, as these interactions are fundamental to the dynamic and functional landscape of cells. Furthermore, dynamic protein interactions are crucial to many biological systems and frequently result in the formation of weak‐encounter complexes, which are difficult to predict by computational methods.

NMR is uniquely suited for the investigation of such weak, transient, and dynamic biomolecular interactions. It provides atomic‐resolution information on biomolecular complexes in various environments, which sheds light on interactions that would otherwise be challenging to identify.^[8]^ In particular, NMR observables are extremely sensitive to noncovalent interactions, with hydrogen bonds and dispersion interactions being the most relevant ones.^[9]^ In addition, NMR can access dynamic timescales covering a very broad range from ns to ms.^[10]^ However, NMR is also limited by the macromolecule size, with large complexes yielding low‐quality spectra in solution‐state NMR and requiring expensive selective labeling of the sample. Solid‐state NMR, in contrast, is not limited by the protein size, but spectral overlap still remains an issue in case of large proteins or complexes thereof. Paramagnetic NMR methods have gained popularity for the analysis of large and complicated assemblies due to the sensitive detection of paramagnetic NMR data and their ability to provide long‐range structural information.[^11^, ^12^] Furthermore, the development of advanced metal ion‐chelating tags for the site‐specific paramagnetic labeling of biomolecules has made paramagnetic NMR accessible to a wide range of applications and systems.^[13]^

Long‐range structural restraints are encoded in the NMR spectra in the form of paramagnetically‐induced residual dipolar couplings (RDCs), pseudocontact shifts (PCSs), and paramagnetic relaxation enhancements (PREs).^[12]^ These restraints provide experimental evidence that validates and adjusts predicted structures, which is extremely useful for refining computational models. Moreover, innovations in dynamic nuclear polarization (DNP) have dramatically enhanced the sensitivity of NMR experiments.[^14^, ^15^] Large protein complexes and biological mixtures that were previously difficult to investigate due to low signal‐to‐noise ratios can now more easily be studied.[^16^, ^17^, ^18^] This enhanced sensitivity enables the capture of detailed structural information even in low‐abundance samples or in cellular systems.[^19^, ^20^, ^21^] While paramagnetic NMR datasets on large protein complexes are usually sparse, the integration with computational modeling tools like AlphaFold[^1^, ^4^] or RoseTTAFold[^2^, ^5^] represents a promising approach to advance their structure determination. Overall, paramagnetic NMR offers a rich toolbox for exploring biomolecular interactions that can validate, refine, and complement computational models, leading to a more complete understanding of biomolecular structures and dynamics.

In this perspective, we review applications of paramagnetic NMR for structural studies of biomolecular complexes. After a short introduction of the theoretical background of paramagnetic NMR effects and an overview of available metal ion‐binding tags, advanced implementations of these methods for studying biomolecular complexes in the solution as well as in the solid state are discussed. We note that paramagnetic tags are a powerful tool for distance measurements also by EPR spectroscopy and refer the reader to recent reviews articles.[^22^, ^23^] We aim to not only summarize past and existing applications, but also highlight the tremendous potential that paramagnetic structure restraints have for guiding molecular modeling methods like AlphaFold and how these synergies can be exploited in the future.

## Toolbox of Available Paramagnetic NMR Methods

**Figure 1:**
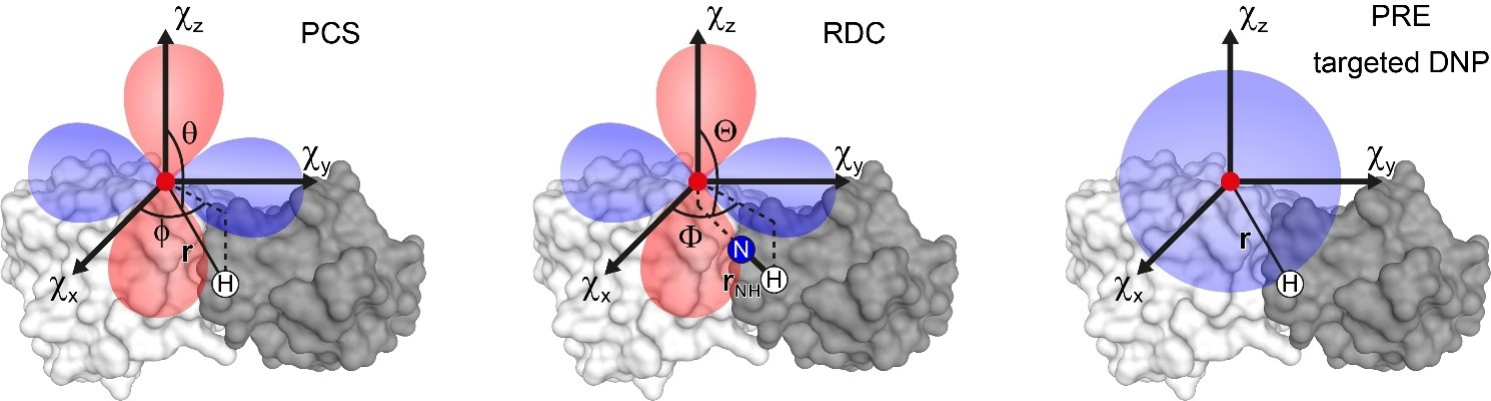
Schematic representation of the different paramagnetic NMR effects that can be used to characterize the structure of biomolecular complexes ‐ PCS, RDC, PRE, and targeted DNP. The PCS and RDC are caused by metal ions with an anisotropic magnetic susceptibility as are illustrated by hypothetical isosurfaces. The PRE and targeted DNP affect the signal intensity of nuclear spins inside a volume of effectively spherical shape around the metal ion.

Dipolar interactions between nuclear spins and unpaired electrons of paramagnetic centers can be detected over long distances due to the much larger gyromagnetic ratio of the unpaired electron spins compared to those of nuclear spins. Because of that, paramagnetic effects tend to be easily detectable in the NMR spectrum, e. g. in the form of large chemical‐shift changes or signal linewidth changes, and provide valuable long‐range structural information. The most informative paramagnetic effects for the study of biomolecular interactions are schematically shown in Figure 1 and briefly described below. For a detailed theoretical description of the different effects see previous articles.[^18^, ^24^, ^25^, ^26^, ^27^]

### Paramagnetic Effects Caused by Magnetic Susceptibility Anisotropy

The dipolar coupling between the unpaired electrons of the metal center and the nuclei leads to a shift of their resonance frequencies which is called the isotropic PCS. Note, that in solids also an anisotropic component exists, similar to the chemical‐shift anisotropy (CSA), which manifests itself in the NMR spectra as magic‐angle spinning (MAS) sideband patterns.^[25]^ PCSs appear in the NMR spectra as chemical‐shift changes with varying signs relative to a diamagnetic reference and can have a remarkably long range of 50 Å and even more.^[28]^ This is because the PCS scales with the inverse third power of the distance between the unpaired electron and the nucleus‐of‐interest.^[11]^ The PCS requires that the magnetic susceptibility tensor of the paramagnetic metal ion is anisotropic. This is the case for several lanthanide ions (e. g., Tb^3+^, Tm^3+^, Dy^3+^, Yb^3+^) and some transition metal ions with significant spin‐orbit coupling (e. g., Co^2+^, Ni^2+^). The PCS also depends on the relative orientation of a nucleus with respect to the metal ion. This spatial information can be exploited in multiple ways to characterize the structure and dynamics of biomacromolecules.^[27]^ PCSs can be used e. g. as structural restraints for structure prediction and structure refinement, as a sensitive measure of protein dynamics, and as a means to reduce the spectral overlap in an NMR spectrum by an increase in chemical‐shift dispersion. Due to its strong angular dependence, the PCS is very sensitive to motions of the metal ion center. Consequently, rigid lanthanide binding tags have been developed over the years to tightly anchor the metal ion center within the biomolecule allowing to further push the long‐range behavior of PCSs.[^28^, ^29^, ^30^, ^31^]

Metal ions with anisotropic magnetic susceptibilities also cause weak alignment of the tagged biomolecule in the external magnetic field, leading to the observation of RDCs between nuclear spins. RDCs offer long‐range angular restraints that can inform on the relative orientation of molecular entities within a common alignment frame and can be used to refine the binding interface of molecular binding partners.^[32]^

### Paramagnetic Relaxation Enhancements

Paramagnetic metal ions also give rise to PREs, which lead to a broadening of the NMR signals and thus changes in peak intensities in a distance‐dependent manner. The PRE decreases with the inverse sixth power of the distance between the paramagnetic center and the nucleus. In contrast to liquid samples, there is however no overall molecular tumbling in solid samples, leading to the absence of the Curie spin relaxation mechanism and its contribution to the PRE in solids.^[33]^ This helps to improve the efficiency of polarization transfer schemes due to the longer coherence lifetimes. The long‐range nature renders PREs a useful tool to provide distance restraints and to detect conformational changes that position the nucleus at different distances from the paramagnetic tag.^[34]^ PREs proved particularly useful as restraints for the structure determination of large protein complexes^[35]^ and for the illumination of minor, “invisible” states present in a biomolecular interaction.^[36]^

As an alternative or complement to spin‐labels, PREs can also be obtained in the presence of soluble paramagnetic probes in solution, which are referred to as solvent PREs (sPREs). An advantage of this method lies in the simple sample preparation protocol. Small, soluble paramagnetic probes, also referred to as co‐solutes, such as organic radicals or metal chelate complexes, are added to the sample without the need for any chemical modification of the biomolecule. sPREs provide information on molecular surfaces and changes in surface exposure upon biomolecular complex formation.^[37]^

### Targeted DNP

As explained above, DNP can effectively increase the sensitivity of NMR by transferring the several orders of magnitude larger thermal polarization of electron spins to surrounding hyperfine‐coupled nuclear spins.^[38]^ This process is typically driven by microwave irradiation of polarizing agents (PAs) added to the solvent during the NMR experiment in the form of stable radicals or suitable paramagnetic metal ions.^[39]^ If the thus hyperpolarized nuclear spins are subject to sufficient homonuclear coupling in a solid, spin diffusion will transport the polarization away from the PA, causing a uniform NMR signal enhancement of the whole sample. The advantage of this approach is that the NMR spectrum may be sensitivity‐enhanced with only minimal paramagnetic perturbation of the resonances.[^14^, ^17^, ^18^]

**Figure 2:**
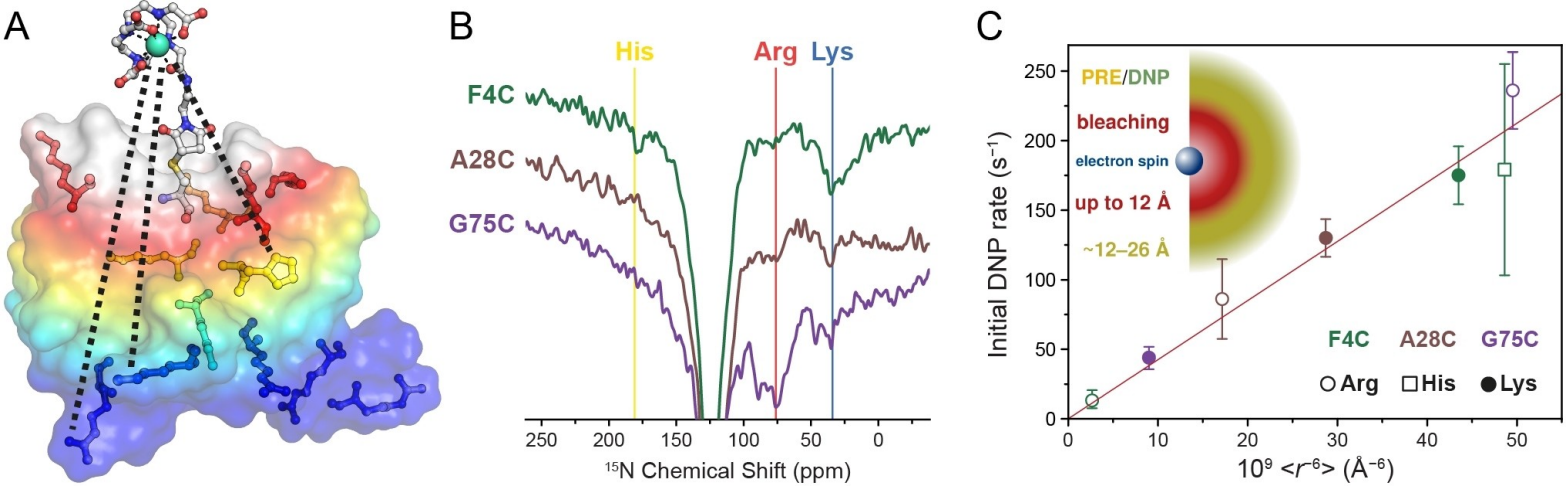
Targeted DNP on Gd^3+^‐labeled ubiquitin. (A) Graphical depiction of the experimental system. The Gd^3+^‐DOTA tag and the position of the amino acid side chains with resolved ^15^N signals (Lys, Arg, His) are shown. The colors indicate different distance ranges: red ‐ up to 12 Å, yellow ‐ 12–25 Å, blue ‐ larger than 25 Å. (B) Direct ^15^N DNP‐enhanced spectra of all three Gd‐labeling positions. Resonance positions for the analyzed side chain signals are highlighted by vertical lines. (C) Plot of the experimentally measured initial DNP rate versus the effective (inverse) Gd^3+^−^15^N distance calculated from computational structure models as is described in the original publication. A linear regression fit through the origin is shown in red. Spin pairs with a distance below the cutoff of 12 Å are excluded, because these pairs are expected to be situated within the bleaching volume of Gd^3+^, while ^15^N in a distance range between 12–26 Å are contributing to the detected signal; this situation is depicted in the inset. Adapted from Heiliger et al.^[44]^ with permission from the PCCP Owner Societies.

In order to focus the DNP effect at a specific site and collect structural information on a biomolecule‐of‐interest, targeted DNP approaches have been developed. In targeted DNP, the polarization is not generated homogeneously throughout the whole sample, but created locally at the target site by paramagnetic tags.[^40^, ^41^] This introduces spectral selectivity as the target site can be specifically hyperpolarized while the unwanted background remains at or near thermal polarization. Care has to be taken, that the generated magnetization is not dispersed from the target site by spin diffusion but remains close to the tagging site. This can be achieved either by full deuteration of the surrounding matrix/environment while the ^1^H spins at the target site are DNP‐enhanced,^[42]^ or by direct DNP of nuclei with a small gyromagnetic ratio and/or low spin concentration.^[43]^ In the latter case, spin diffusion may be sufficiently retarded with respect to the DNP transfer, allowing for distance‐dependent effects to be observed.^[44]^ As is shown in Figure 2, the DNP buildup rate (i. e., the rate with which polarization is transferred directly from the electron spin to the nucleus observed by NMR) is the most promising observable as it scales with the inverse sixth power of the electron‐nuclear distance. Counterintuitively, the DNP enhancement of individual nuclei is not suited as a distance measure because the PRE underlies the same scaling as the DNP transfer rate, leading to distance‐independent polarization enhancement factors around the target site.[^44^, ^45^] In contrast to relatively sparse low‐γ nuclei, even if ^1^H is locally hyperpolarized by a PA tag, the strong homonuclear couplings quickly cause a uniform distribution over all connected proton spins. Nevertheless, site‐specific information about the target site may still be obtained by analyzing the signal bleaching induced by the paramagnetic label while benefiting from increased sensitivity.[^46^, ^47^] Up to the time of writing, (semi−)quantitative distance information by targeted DNP has only been achieved for dilute and low‐γ spins such as ^15^N. However, special methods like SelDNP^[47]^ and SCREAM‐DNP^[48]^ can extend the application range to ^1^H as well.

## Paramagnetic Labeling

**Figure 3:**
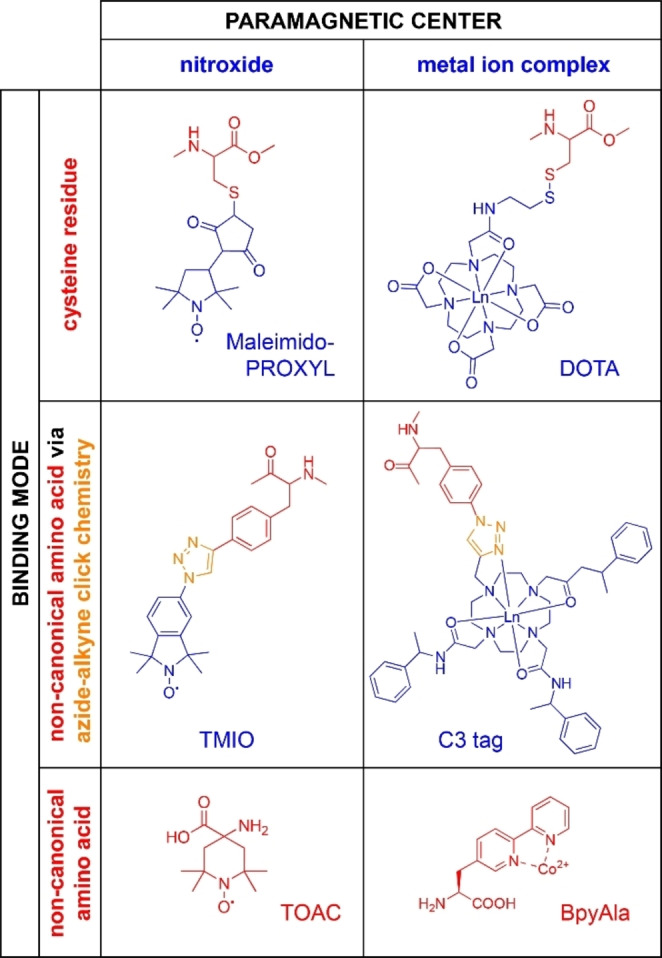
Overview of paramagnetic tagging strategies. Six exemplary tags or nitroxide or metal‐ion bearing non‐canonical amino acids are shown: PROXYL (2,2,5,5‐tetramethyl‐1‐pyrrolidinyloxy), DOTA (1,4,7,10‐tetraazacyclo‐dodecane‐1,4,7,10‐tetra‐acetic acid), TOAC (2,2,6,6‐tetramethyl‐N‐oxyl‐4‐amino‐4‐carboxylic acid), BpyAla (bipyridylalanine),^[58]^ C3,^[59]^ and TMIO (1,1,3,3‐tetramethylisoindolin‐2‐oxyl).

Paramagnetism can exist naturally in biomolecules or can be introduced artificially using different strategies which are shortly described here (see Figure 3). For comprehensive reviews summarizing paramagnetic spin tags see[^13^, ^49^]. Metalloproteins containing paramagnetic metal ions, such as the protein concanavalin A containing Mn^2+^,^[50]^ can be directly studied by paramagnetic NMR. In some instances, the naturally occurring diamagnetic metal ion can be substituted for a paramagnetic ion with a similar radius and the same charge. In suitable cases, an endogenously bound metal ion polarizing agent may also enable targeted DNP.^[51]^ Similarly, the flavin mononucleotide (FMN) cofactor of flavodoxins could be used to selectively hyperpolarize the protein, taking advantage of the natural semiquinone radical state of FMN.^[52]^ Inspired by the ability of substituting metal ions in metalloproteins, so‐called lanthanide binding peptides were developed, which can be fused to the N‐ or C‐terminus of proteins.[^53^, ^54^] Subsequently, lanthanide binding peptides for the insertion into loops[^55^, ^56^] or for two‐point attachment^[57]^ were developed, which reduced the tag mobility and enhanced the paramagnetic effects.

If a natural metal center is lacking, paramagnetic spin labels can be introduced by site‐directed spin labeling. Nitroxides are the most frequently used paramagnetic spin labels for the generation of PREs due to their small size and long electronic relaxation time. MTSL (*S*‐(1‐oxyl‐2,2,5,5‐tetramethyl‐2,5‐dihydro‐1H‐pyrrol‐3‐yl)methyl methanesulfonothioate) is the most popular nitroxide and can be attached to biomolecules via free thiol groups e. g. of protein cysteine residues. Similar spin labels are based on TEMPO (2,2,6,6‐tetramethyl piperidin‐1‐oxyl) or PROXYL (2,2,5,5‐tetramethylpyrrolidine‐N‐oxyl nitroxide) radicals, and can be introduced using maleimido linkers for a Thiol‐Michael addition coupling reaction.

To achieve site‐selectivity, this approach requires the removal of all solvent‐exposed cysteines and the reintroduction of a single cysteine mutation. However, endogenous cysteine residues are inherently important to the overall three‐dimensional fold, stability, and function of a protein and in some cases, cannot be removed. An orthogonal strategy is the use of genetically encoded non‐canonical amino acids that either bear a paramagnetic center or a reactive group for the attachment of a metal ion‐chelating group via e. g. click‐chemistry.^[60]^ For example, Loh et al.^[59]^ reported the site‐specific incorporation of the non‐canonical amino acid *p*‐azido‐*L*‐phenylalanine and ligation with alkyne‐bearing lanthanide tags via a copper‐catalyzed azide‐alkyne cycloaddition click chemistry reaction. Another frequently utilized framework for paramagnetic labeling are lanthanide‐chelating tags, for instance those based on DOTA (1,4,7,10‐tetraazacyclododecane‐1,4,7,10‐tetraacetic acid).^[61]^ Also, direct incorporation of the nitroxide‐bearing unnatural amino acid TOAC into the peptide backbone has been reported.^[62]^

When covalent labeling of the biomolecule should be avoided, paramagnetic co‐solutes offer a possibility. The co‐solutes are simply added to the sample and will distribute around the biomolecule of interest. They should be highly soluble and lack any specific, strong interactions with functional groups on the biomolecule. An exception are cases in which one aims to study electrostatic surface properties of biomolecules, such as the distribution of ions^[63]^ or local electrostatic fields in biomolecules,[^64^, ^65^] for which charged paramagnetic molecules are useful. In addition to molecular oxygen (dioxygen),^[66]^ which can be used to determine the immersion depths of membrane proteins,^[67]^ polar or charged derivatives of TEMPO^[68]^ and PROXYL^[69]^ are commonly used. Gd^3+^ chelates induce the strongest solvent PRE effects and Gd(DTPA‐BMA) (diethylenetriamine pentaacetic acid‐bismethylamide) is the most commonly used probe of all Gd^3+^ chelates.^[70]^ Charged probes, such as Carboxy‐PROXYL or Gd(DOTA)^−^, have been exploited less often, but offer a sensitive tool to determine near‐surface electrostatic potentials of biomolecules.[^64^, ^71^, ^72^]

## Applications of Paramagnetic NMR Techniques for Probing Biomolecular Interactions

**Figure 4:**
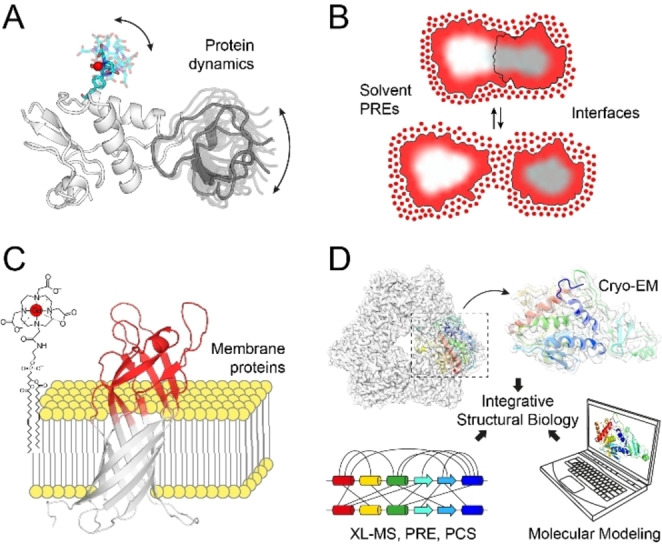
Application areas of paramagnetic NMR for the study of biomolecular interactions. (A) Detection of dynamic protein‐protein interactions, (B) detection of protein interfaces using solvent PREs, (C) detection of protein interactions with other biomolecules, and (D) integration with other structural biology techniques for the structure determination of large complexes.

Paramagnetic NMR techniques offer a versatile toolbox for the investigation of different biomolecular interactions and systems (Figure 4). In the following, we focus on four selected application areas where paramagnetic NMR plays an important role now and in the future, namely the characterization of dynamic protein‐protein interactions, the identification of protein‐protein interfaces, the investigation of protein interactions with other biomolecules, such as small‐molecule ligands, nucleic acids or membranes, as well as the use of paramagnetic NMR data for integrated structural biology studies and biomolecular modeling.

### Study of Dynamic Protein‐Protein Interactions

Paramagnetic NMR data are very sensitive to even small structural changes, which renders them a unique tool to study the dynamic behavior of biomolecular interactions. In terms of PCSs, relative motions between the metal tag and the biomolecule (Figure 4A) will reduce the apparent magnitude of the Δ*χ*‐tensor, which will be indicated by decreased PCSs. A dynamic protein‐protein complex that could be elucidated with the help of PCSs has been provided by the synaptotagmin‐1‐SNARE complex.^[73]^ Lanthanide C2 tags were attached to the SNARE complex, consisting of syntaxin‐1 and SNAP‐25, but only two of ten tagging sites tested yielded sizeable PCSs. Nevertheless, the PCS data as input to docking calculations yielded a molecular binding model in which the basic residues on the synaptotagmin‐1 C2B domain interact with a polyacidic region on the SNARE complex.

Conformational exchange may impede the NMR detection of biomolecular complexes due to spectral overlap and peak broadening. This renders standard NMR approaches used for more rigid protein complexes impossible. However, even sparse paramagnetic NMR datasets in combination with model building can generate informative insight as demonstrated for the ensemble of closed and open forms of the dengue virus NS2B−NS3 protease.^[74]^ Small‐molecule inhibitors were shown to trigger a closed conformation of the protease while the open form was barely populated in solution. Another example is the conformational changes observed in the 47 kDa ATP‐driven ligase MurD, studied by Saio et al.,^[75]^ revealing a semi‐closed state that may regulate enzyme substrate binding. If a protein conformation of interest is present in low abundance, chemical exchange saturation transfer (CEST) experiments can be used to measure PCSs of the lowly populated state. This was demonstrated for the complex of actin regulating kinase 1 peptide (Ark1p) and the actin binding protein (Ab1p) SH3 domain, for which PCSs of the bound state could be detected even in the presence of only 3 % peptide.^[76]^ An analogous approach to study conformational transitions is by means of Carr‐Purcell‐Meiboom‐Gill (CPMG) relaxation dispersion experiments. The combination of PCSs and CPMG offers advantages because the PCSs can magnify the amplitude of the relaxation dispersion profiles, which improves the detection of the exchange process. Using this method, the structure and kinetics of high‐energy states of adenylate kinase in catalysis could be studied.^[77]^ This work also highlighted the importance of improving the rigidity of lanthanide tags to minimize relaxation dispersion from tag movements.[^77^, ^78^]

Amongst the paramagnetic NMR effects, PREs offer outstanding opportunities for the characterization of sparsely populated conformational states. Those states cannot be detected by other structural biology methods but are accessible to PREs if the minor state undergoes rapid exchange with the major state (with exchange timescales <250 μs) and if the distance between the paramagnetic center and some of the nuclei measured is shorter in the minor state than in the major state. Because of the r^−6^ dependence, a nucleus in close proximity to a paramagnetic center will exhibit an extremely large PRE, which will dominate the overall PRE measured for that nucleus, even if it constitutes a minor percentage of the total population. This permits structural characterization of lowly populated and transient conformational states. For example, PRE data were used to elucidate the DNA binding mode of DNA transcription factors,^[79]^ revealing transient interactions with non‐specific DNA sequences that proceed and facilitate the formation of the specific complex. Furthermore, PRE data have provided molecular details on protein‐protein encounter complexes,[^80^, ^81^, ^82^] on the initial steps of protein oligomerization,^[83]^ and on the interaction sites of small proteins sampled in supramolecular complexes.^[84]^

To study protein conformational changes also by the DNP mechanism, a creative method has been invented by Wylie et al.^[85]^ Two single nitroxide labels were either attached at the two subunits of a dimeric complex or at two distant positions along the primary sequence of a monomeric protein. The cross‐effect DNP mechanism was only enabled, when the two nitroxide labels came into inter‐ or intramolecular close contact, respectively, upon dimer formation or folding of the protein, while no DNP enhancement was observed when the nitroxides remained separated.^[85]^

### Detection of Interaction Surfaces

Besides applications in NMR signal enhancement^[86]^ and NMR spectral editing,^[87]^ the sPRE method has been used in several ways to gain information about the structure and dynamic behavior of biomolecular complexes. sPREs were used to map the binding interfaces between the constituents in protein complexes.^[88]^ Residues located in protein‐protein interaction interfaces or in the protein interior are shielded from the sPRE probe and experience a weak PRE, whereas residues located at solvent accessible surfaces experience a strong PRE. By measuring PREs for the bound complex and for the separate unbound proteins, the residues located in interfaces can be distinguished from those buried in the protein interior (Figure 4B). Importantly, sPREs are very sensitive to weak and dynamic interactions. Johansson et al.^[89]^ used them to distinguish between weak, specific interactions and transient, non‐specific interactions. In studying the self‐association of human growth hormone (hGH), the authors observed that the sPREs of residues involved in non‐specific interactions increase linearly with higher hGH concentration while the sPREs of residues involved in specific interactions decrease at higher hGH concentrations, because access of the paramagnetic Gd(DTPA‐BMA) probe to the residues in the interaction surface was impeded.^[89]^

Recently, Penk et al.^[71]^ demonstrated that the sPRE method can also be used to map the binding interface of small, drug‐like peptides on proteins. By evaluating the differential sPREs of peptide‐bound and free growth factor receptor‐bound protein 2 (Grb2) in the presence of different PROXYL co‐solutes, the authors could determine the peptide binding interface on Grb2 in perfect agreement with the X‐ray structure pose.

sPREs can also be measured in the solid state, as shown for α‐spectrin SH3 doped with paramagnetic Cu^2+^.^[90]^ An overall acceleration of the relaxation rates could be observed that allowed for faster acquisition of solid‐state NMR spectra. The PREs were not uniformly distributed throughout the protein chain, but water‐accessible residues experienced a stronger PRE than those further away from the surface. This method of probing the water‐accessible surface of a protein can even be applied in a qualitative way if the protein structure is unknown. Similarly to the study on α‐spectrin SH3, protein‐solvent interfaces of human PrP23‐144 amyloid fibrils were determined from ^15^N longitudinal PREs, confirming that the structure is composed of two protofilaments.^[91]^

### Detection of Interactions with other Molecules

Many proteins function in complex with other biomolecules like membranes or nucleic acids or can be targeted by small molecule ligands or drugs. Also for these systems site‐directed labeling strategies were developed, giving access to site‐specific structural information.

#### Membrane Proteins

Paramagnetic NMR, in particular the PRE, is a widely used tool to determine the association of peptides and proteins with lipid membranes. A small amount of spin‐labeled lipid molecules is usually incorporated into the lipid bilayers, which induces PREs on the protein when it interacts with the membrane (Figure 4C). Thus, the protein orientation on the membrane and membrane partitioning can be inferred.^[92]^ PREs combined with other NMR data have offered comprehensive, residue‐specific structural information for several membrane proteins.[^93^, ^94^] For example, Jirasko et al.^[95]^ used a synthetic lipid with a Gd^3+^‐chelating headgroup to identify regions on the multidomain NS5A AH‐linker‐D1 protein that are in close proximity to the membrane. The PRE solid‐state NMR data revealed the presence of NS5A dimers in the membrane that differed significantly from the crystal structure and served as basis to model the mode of action of NS5A inhibitors. Noteworthy, these experiments can be complemented by measurements using paramagnetic co‐solutes (e. g. gadodiamide), offering a means to determine the orientation and immersion depth of transmembrane peptides within membrane mimetics.[^96^, ^97^]

One way to enhance the usually low NMR signal of membrane proteins is via targeted DNP from spin‐labeled lipid molecules, which could achieve higher DNP enhancements than experiments with soluble, non‐lipid anchored radicals.[^98^, ^99^, ^100^] Signals from bacterial cell walls can also be either completely bleached or drastically enhanced because the biradical TOTAPOL (1‐(TEMPO‐4‐oxy)‐3‐(TEMPO‐4‐amino)propan‐2‐ol) tends to accumulate in peptidoglycan. This makes in‐cell DNP possible because background signals can be suppressed.^[101]^

#### Small Molecule Ligands

The long‐ranging nature of paramagnetic NMR makes it well possible to study the binding pose of small‐molecule ligands on proteins using tags that are attached at remote sites in the protein. An early example of this reporter group strategy can be found in Zhuang et al. who attached a lanthanide binding tag to galectin‐3′s C‐terminus and used PCSs and RDCs induced on the ligand lactose to determine its binding pose.^[102]^ Similarly, Künze et al. measured PCSs on a heparin tetrasaccharide interacting with the protein IL‐10 and used a combined PCS‐restrained docking and MD simulation approach to optimize the ligand's position and orientation.^[103]^ Guan et al. studied interactions between FKBP13 and a fragment hit molecule using PCS data generated via a two‐armed lanthanide tag to accurately localize the ligand inside the binding site.^[104]^

The relatively high abundance of ^19^F atoms in drug molecules and their absence in biomolecules makes ^19^F a favorable nucleus for paramagnetic NMR. Zimmermann et al. showed that the position of different ligands of carbonic anhydrase II can be accurately determined using PCSs measured from one‐dimensional ^19^F spectra.^[105]^ Gao et al. used ^19^F CEST experiments to determine bound‐state ^19^F PCSs of inhibitors of the BRM bromodomain.^[106]^ This approach can be helpful to characterize ligands in the intermediate NMR exchange regime. Other spectroscopic reporter groups, e. g. tert‐butyl groups, are also suited for measurement of ligand PCSs, as demonstrated by Chen et al. for a tightly binding lead compound of the dengue virus NS2B−NS3 protease.^[107]^

Site‐selective information on protein‐ligand complexes could also be obtained using targeted DNP by attaching the polarizing agent TOTAPOL to the Bac peptide which binds Bcl‐xL,^[108]^ or by using a TOTAPOL‐trimethoprim conjugate targeting dihydrofolate reductase.^[42]^

#### Protein‐Nucleic Acid Complexes

Paramagnetic NMR can aid in the structure determination of protein nucleic‐acid complexes, as well. Wu et al. developed a DNA labeling tag which targets phosphorothioate nucleotides and can generate sizable PCSs in DNA.^[109]^ Strickland et al. developed an approach to measure PCSs in RNAs via a paramagnetically tagged reporter protein, U1A.^[110]^ The fact that the U1A binding motif can be introduced in hairpin and loop structures in RNA molecules makes this method generally applicable to the characterization of the larger RNA complexes. Paramagnetic solid‐state NMR has also facilitated the determination of the structure of a protein‐RNA complex.^[111]^ The protein was spin‐labeled with a nitroxide‐tag and PRE‐based intermolecular distance restraints between the protein and RNA were used for structure calculation supplemented by chemical‐shift perturbations as a second source of restraints.

### Integration of Paramagnetic NMR with other Structural Biology Techniques

Resolving large biomolecular complexes is often not possible using a single technique but requires integrating data from multiple sources. In this regard, NMR is an important part of the toolbox of integrative structural biology, in addition to techniques like cryo‐EM, cross‐linking mass spectrometry and computational modeling (Figure 4D). Integrating these additional biophysical data and techniques can help to close the gaps left by sparse paramagnetic NMR datasets and can improve the accuracy of the structural model. An example is found in Cerofolini et al. who used small‐angle X‐ray scattering (SAXS) and PCS data to analyze matrix metalloproteinase‐1 conformations.^[112]^ Furthermore, Strickland et al. used an integrative structural biology approach, including PCS measurements, SAXS, X‐ray, and computational modeling, to identify the structure of the NPr:EINtr complex,^[113]^ which revealed the mechanism responsible for preventing cross‐reaction with a paralogous phosphotransferase enzyme complex.

**Figure 5:**
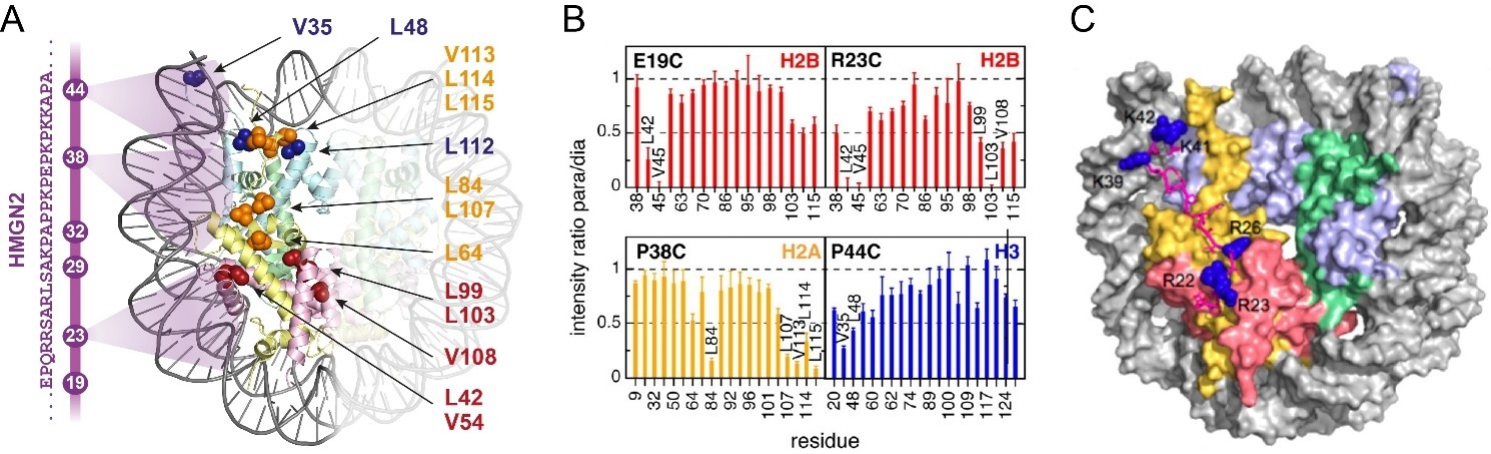
PRE data‐guided structure determination of the HMGN2‐nucleosome complex. (A) The positions of the spin label sites in the nucleosome binding domain of HMGN2 are shown on the left side. Residues in histone proteins with large PREs are labeled and their side chains are depicted with spheres in the nucleosome structure (PDB ID 2PYO). (B) Methyl group signal intensity changes of residues in histones H2A, H2B, and H3 induced by HMGN2 labeled with Mn^2+^‐EDTA at different positions (E19C, R23C, P38C and P44C). (C) Docking model of the HMGN2‐nucleosome complex calculated using PRE distance restraints. The nucleosome is presented as a surface, the HMGN2 nucleosome binding domain is drawn with sticks and the side chains of positively charged residues are indicated with blue spheres. Panels A–C were reproduced from Kato et al.^[113]^, copyright 2011, National Academy of Sciences, USA.

PREs have been instrumental for the structure elucidation of several large protein complexes, in combination with other NMR and structural biology techniques. For example, the structure of the 210 kDa *E.coli* SecA complex bound to a secretory signal peptide could be determined with the help of approximately 160 intermolecular PREs collected from two spin labeling sites on the LamB signal peptide.^[114]^ Because the crystal structure of apo SecA was known, docking could be used to develop a structural model of the SecA‐signal peptide complex. This provided insight into the recognition mode and the conformational changes that SecA undergoes to accommodate the signal peptide. By using a similar strategy of combining CSP and PRE data on selectively methyl‐labeled samples with protein docking, Kato et al. determined the structure of the 230 kDa nucleosome core particle (NCP) bound to HMGN2 protein (Figure 5).^[115]^ HMGN2 plays roles in various processes, including transcription, DNA repair, chromatin remodeling, and histone modification.^[116]^ The structural model showed that HMGN2 binds to the NCP in an extended form and that it bridges sites in the H2A−H2B histone dimer and the nucleosomal DNA (Figure 5).

**Figure 6:**
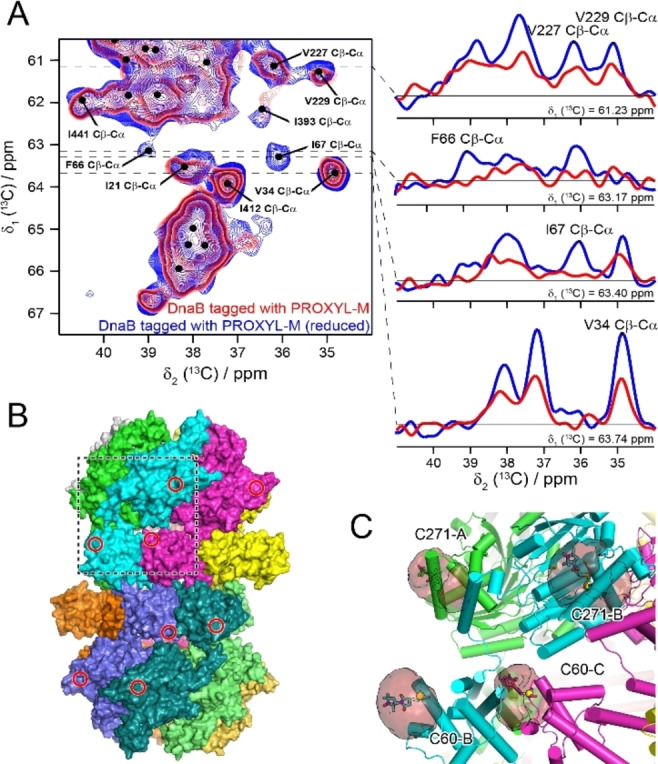
Solid state PRE measurements on the *H. pylori* DnaB complex. (A) 2D ^13^C−^13^C DARR spectra of diamagnetic and paramagnetic DnaB tagged with a PROXYL‐M tag. Some selected traces along the F2 dimension are shown on the right side. (B) Structural model of the DnaB complex with the 12 subunits shown in different colors. The cysteine labeling sites at C60 and C271 are shown for the four subunits in the front, which are colored cyan, magenta, light blue and deep teal, respectively. (C) Zoom in view showing four different tagging sites located on three adjacent DnaB subunits. The space sampled by the O1 atom of PROXYL‐M is shown as red transparent volumes. Reproduced from Zehnder et al.^[118]^ under permission of Creative Commons Attribution 4.0 International License (https://creativecommons.org/licenses/by‐nc‐nd/4.0/).

Two other impressive examples of supramolecular complexes, where the use of PRE distance restraints was instrumental to determine the structure, include the 390 kDa box C/D ribonucleoprotein complex,^[117]^ resolved by the Carlomagno lab, and the 650 kDa ClpB‐DnaK complex,^[118]^ elucidated by the Kay lab. These studies have demonstrated the effectiveness of combinatorial protocols employing selective labeling schemes, paramagnetic NMR, complementary biophysical or biochemical experiments and computational modeling to determine the structures of even large protein assemblies. Paramagnetic NMR can often work in synergy with complementary distance information obtained by EPR on the same samples. One recent example is the structural characterization of the ATP‐hydrolysis transition state of the bacterial DnaB helicase, in which EPR allowed localizing the ATP metal ion cofactor in the active site of the enzyme and solid‐state NMR enabled to unravel the nucleotide binding modes.^[119]^ In general, the combination of paramagnetic solid‐state NMR with EPR offers promising potentials for biomolecular structure characterization. This was demonstrated for the bacterial DnaB helicase from *H. pylori*.^[120]^ EPR on the Gd^3+^‐tagged 708 kDa ATP‐driven motor protein confirmed the dodecameric quaternary structure of the complex; additionally, PREs measured on the either nitroxide or Gd^3+^‐tagged protein confirmed the protein tertiary structure of DnaB (Figure 6). This was critical in order to validate and further improve the only available, low‐resolution X‐ray structure of DnaB. In a second study, the DnaB protein was trapped in the ATP‐binding transition state with the metal ion cofactor exchanged from Mg^2+^ to paramagnetic Co^2+^ or Mn^2+^.^[121]^ DnaB samples for solid‐state NMR were prepared by sedimentation using an ultracentrifuge. This sample preparation technique avoids the time‐consuming and sometimes even impossible crystallization step,[^122^, ^123^] which has been particularly convenient for the studied DnaB protein.^[124]^ PREs were identified in different multidimensional NMR spectra and translated into long‐range distance restraints. These restraints enabled the de‐novo localization of the positions and Δ*χ*‐tensors of the six metal ions in DnaB in case of Co^2+^.

### Integration of Paramagnetic NMR with Computational Modeling

Paramagnetic NMR datasets are often sparse and, if considered on their own, fail to provide enough resolution, accuracy, unambiguity, and coverage to determine biomolecular structures. Integrating computational modeling with experimental data is a powerful approach to supplement missing structural information with atomic detail. To expand the range of applications of paramagnetic NMR, computational tools for PCSs, RDCs, and PREs were implemented in macromolecular modeling software such as HADDOCK^[125]^ and Rosetta.^[ 126–129]^ HADDOCK implements different NMR data types for docking^[130]^ and can also be used to predict the structure of protein‐nucleic acid^[131]^ and protein‐peptide complexes.^[132]^ A similarly rich set of protocols for different prediction tasks is offered by the Rosetta software suite.^[133]^ A recent review on Rosetta's NMR modeling tools can be found in Koehler Leman et al.^[134]^

Despite the recent breakthroughs achieved by AI‐based modeling methods like AlphaFold, predicting protein complex structures remains difficult, especially when there is significant conformational change in one or more binding partners. Multimer prediction is more challenging than monomer prediction because residue co‐evolutionary signals between proteins are much weaker than within monomers or are missing at all.^[135]^ Modifications to the AlphaFold sampling protocol can enhance prediction performance, but the success rate for dimeric complexes is usually not higher than 60–70 %^[136]^ and decreases further for complexes with more chains.^[137]^ In this case, paramagnetic NMR data can help to resolve ambiguities in the structure prediction and guide the computational modeling. The recently published method AlphaLink,^[138]^ a modified version of AlphaFold2, incorporates distance restraints into the AlphaFold2 network and offers improved performance on difficult targets, such as proteins with a limited number of homologous sequences. While AlphaLink was originally developed for MS cross‐linking data, the authors note that it can also make use of other distance information, e. g. PRE and NOE data. The combination of AlphaLink with PREs promises to become an effective approach for predicting large protein complexes from sparse NMR data.

Another example how paramagnetic NMR data and AlphaFold can complement each other is in the modeling of complexes which undergo conformational changes. These changes cannot be reliably predicted with the standard AlphaFold2 protocol, but can be visualized by NMR. An interesting study showing a combined AlphaFold and NMR approach has been recently reported by the Montelione group.^[139]^ The authors chose a conformer‐selection strategy in which a large collection of plausible protein models is first generated using an AlphaFold‐enhanced sampling protocol,^[140]^ followed by the selection of a smaller set of conformers that best explains the experimental NMR data. The multistate‐ensemble structure satisfied the NMR data better than the previously published NMR structure,^[141]^ which was calculated directly from the NMR restraints, showing the advantage of this method.

## Outlook

The herein discussed applications of paramagnetic NMR reveal that the relevance of this method for integrated structural biology is expected to further grow in the next years. In combination with targeted DNP, the study of biomolecular complexes directly in their cellular environments could be taken into the focus.^[21]^ Furthermore, advances in paramagnetic spin‐labeling of nucleic acids will allow probing transient interactions in protein‐nucleic acid complexes for instance in the strongly emerging field of liquid‐liquid phase separation involving RNA‐binding proteins.^[142]^ These applications will strongly benefit from advances in instrumentation. For instance, proton‐detected MAS experiments at ever higher spinning frequencies (>160 kHz) will further improve spectral resolution in proton‐detected solid‐state NMR spectra enabling the investigation of biomolecular assemblies,[^143^, ^144^] which are outside the possibilities of NMR in solution. The development of novel extraordinary rigid paramagnetic tags will further push the detection limit of molecular distances. This will also significantly simplify NMR resonance assignments in case of otherwise crowded NMR spectra, since the chemical‐shift dispersion will be increased. We could envision that this might also decrease the coherent contribution to the proton NMR linewidths in solids. A particular challenge in paramagnetic NMR investigations often is to disentangle intra‐ from intermolecular effects. The technique of sedimentation might offer a solution here due to the statistical distribution of protein monomers in a sediment.[^120^, ^145^] In addition, novel sample preparation protocols for solid‐state NMR, such as the immobilization of proteins in suitable matrices,^[146]^ might broaden the applications of paramagnetic solid‐state NMR.

## Conflict of Interests

The authors declare no conflict of interest.

1

## Data Availability

Data sharing is not applicable to this article as no new data were created or analyzed in this study.

## Footnotes

n1
https://www.rcsb.org, accessed on 17. 10. 2024.
